# The Interaction Efficiency of XPD-p44 With Bulky DNA Damages Depends on the Structure of the Damage

**DOI:** 10.3389/fcell.2021.617160

**Published:** 2021-03-11

**Authors:** Irina Petruseva, Natalia Naumenko, Jochen Kuper, Rashid Anarbaev, Jeannette Kappenberger, Caroline Kisker, Olga Lavrik

**Affiliations:** ^1^Laboratory of Bioorganic Chemistry of Enzymes, Institute of Chemical Biology and Fundamental Medicine SB RAS, Novosibirsk, Russia; ^2^Rudolf Virchow Center for Integrative and Translational Bioimaging, University of Wuerzburg, Wuerzburg, Germany

**Keywords:** nucleotide excision repair, XPD helicase, DNA damage, protein-DNA interaction, bulky damages recognition, photo-cross-linking

## Abstract

The successful elimination of bulky DNA damages via the nucleotide excision repair (NER) system is largely determined by the damage recognition step. This step consists of primary recognition and verification of the damage. The TFIIH helicase XPD plays a key role in the verification step during NER. To date, the mechanism of damage verification is not sufficiently understood and requires further detailed research. This study is a systematic investigation of the interaction of ctXPD *(Chaetomium thermophilum)* as well as ctXPD-ctp44 with model DNAs, which contain structurally different bulky lesions with previously estimated NER repair efficiencies. We have used ATPase and DNA binding studies to assess the interaction of ctXPD with damaged DNA. The result of the analysis of ctXPD-ctp44 binding to DNA containing fluorescent and photoactivatable lesions demonstrates the relationship between the affinity of XPD for DNAs containing bulky damages and the ability of the NER system to eliminate the damage. Photo-cross-linking of ctXPD with DNA probes containing repairable and unrepairable photoactivatable damages reveals differences in the DNA interaction efficiency in the presence and absence of ctp44. In general, the results obtained indicate the ability of ctXPD-ctp44 to interact with a damage and suggest a significant role for ctp44 subunit in the verification process.

## Introduction

The nucleotide excision repair (NER) pathway removes a wide range of bulky DNA damages with high accuracy. Damages appear as a result of various chemical and physical impacts with most of them being adducts of nitrogenous bases, that cause significant distortions of the regular double-stranded DNA structure. Eukaryotic NER involves about 30 proteins, which sequentially form complexes of different compositions on DNA. To date, significant progress has been made toward our understanding of the multi-stage NER mechanism but an important step in the process, i.e., the damage verification step, is still poorly understood. In particular, the rates of removal of bulky damages vary by orders of magnitude, but the reason for these variations are insufficiently clear so far, although the basic principles of their recognition and the criteria for repair susceptibility are formulated (Mu et al., 2018).

Damage recognition is a key stage of the NER process that largely determines the rate of DNA repair (Schärer, 2013). It can be described as a step-by-step process that includes the primary recognition of damage and its subsequent verification. The XPC-HR23B-Centrin2 complex, which performs primary damage recognition for bulky lesions, is sensitive to distortions of the regular double helix structure and interacts with regions with disturbed and destabilized hydrogen bonds with increased affinity. With respect to a less distorting lesion, i.e., cyclo-butane pyrimidine dimers (CPDs) caused by UV light, the XPC-HR23B-Centrin2 complex requires in addition the UV-DDB complex for damage detection (Compe and Egly, 2016).

According to the generally accepted model of “indirect recognition,” XPC-HR23B does not directly recognize the damage (Min and Pavletich, 2007; Jain et al., 2013; Schärer, 2013), rather the damage topology and induced distortions of the regular DNA structure affect the efficiency of the formation of productive XPC-DNA complexes (Reeves et al., 2011; Mu et al., 2018). The initial recognition stage is followed by the “contact” stage, i.e., verification of the presence of a bulky modification. This is the catalytic stage that involves the ATP-dependent helicase subunits of the TFIIH factor, which display an increased affinity for the XPC-DNA complexes (Araújo et al., 2001). One of the key players in this verification stage is the 5′→3′ helicase XPD. It is assumed that XPD within TFIIH engages with DNA. Subunit p44 directly stimulates XPD’s 5′ to 3′ helicase function. When XPD encounters the damage, its helicase activity is inhibited and XPD is immobilized on DNA, thus finally marking the damage (Naegeli et al., 1993; Coin et al., 2007; Oksenych and Coin, 2010; Buechner et al., 2014; Wirth et al., 2016).

XPD belongs to the SF2B family of helicases and consists of two RecA like helicase domains (HD1 and HD2), an Arch-domain, and a 4Fe4S-cluster domain. A significant understanding of the structural organization and mechanism of the action of human XPD was achieved through the analysis of its archaeal orthologs.

The structural and biochemical analysis of the complex of XPD from the *Thermoplasma acidophilum* protein (taXPD) with DNA in combination with functional mutagenesis made it possible to propose a model of DNA binding in which DNA binds parallel to the HD1 and HD2 domains. HD2 contains a high-affinity ssDNA binding site that might serve as an initial binder. The continuation of the strand toward HD1 leads the DNA to a pore-like feature, which has been suggested to assume the function of a lesion recognition sensor and is formed by HD1, the Arch domain, and the 4Fe4S domain. After passing the pore, DNA may further bind to a basic groove that is formed by HD1 and the 4Fe4S domain (Kuper et al., 2012; Pugh et al., 2012; Constantinescu-Aruxandei et al., 2016). Recently, this model was further validated by a cryo-EM structure, where human XPD assumed a similar orientation with a bound DNA fragment, thus indicating that DNA binding to XPD is highly conserved (Kokic et al., 2019). Based on these analyses, it has been proposed that the rate of DNA unwinding decreases when the damaged unit of the strand interacts with the sensor pocket, i.e., an “immobilization” of XPD takes place at this point. The effect of the damage-induced decrease in the helicase activity was first demonstrated for its yeast homolog Rad3 (Naegeli et al., 1993). Further comparative mutagenesis studies showed that the location of the sensory pocket in human XPD (hXPD) coincided with its location in taXPD (Mathieu et al., 2013). However, some data suggest that bulky damages do not affect the archaeal helicase activity (Rudolf et al., 2009). There may be several reasons for this discrepancy. Despite the similarity of the domain architecture, XPDs from archaea function as isolated monomers, whereas eukaryotic lesion verification requires the presence of the multiprotein complex TFIIH, with XPD being one of the subunits in this complex.

The XPD helicase from the eukaryotic fungus *Chaetomium thermophilum* was recently utilized as a model to study hsXPD. This helicase, like the human protein, functions as a part of the protein TFIIH complex. It has been shown that the activity of ctXPD is tightly regulated by the interaction with the TFIIH subunits ctp44 and MAT1 (Kuper et al., 2014; Peissert et al., 2020). Ctp44 directly activates the ATPase function of ctXPD; meanwhile the presence of ctp44 does not alter the ctXPD affinity for DNA significantly (Kuper et al., 2014).

In this work, we characterized the interaction of ctXPD and the ctXPD-ctp44 complex with model DNA substrates, which contained bulky damages in the translocated strand. The structures of the DNA model lesions are shown in Supplementary Figure 1S and the DNA sequences are listed in Supplementary Table 1S. Our data indicate that the high efficiency of the NER-catalyzed excision of the bulky damage from DNA are associated with a high affinity of ctXPD to the damaged translocated strand. The experiments were performed using DNA that contained photoactivatable bulky damages. Our data show that both ctXPD and ctp44 interacted with the damage in the translocated strand and formed covalent adducts with the damage, which indicates that also the latter TFIIH subunit might assume additional functions at the stage of damage verification.

## Materials and Methods

### Materials

All synthetic oligonucleotides were synthesized in the Laboratory of Biomedical Chemistry (ICBFM SB RAS, Russia). Amidophosphites for nFlu- and nAnt-containing oligonucleotides were synthesized as described (Evdokimov et al., 2013). [γ-^32^P]ATP (3000 Ci/mmol), [α-^32^P]ATP (3000 Ci/mmol) was from the Laboratory of Biotechnology (ICBFM SB RAS, Russia). T4 polynucleotide kinase, Taq polymerase, and T4 DNA ligase were from Biosan (Novosibirsk). Fab(g)-dCTP and Fap-dCTP were synthesized as described (Dezhurov et al., 2005; Safronov et al., 1997). Recombinant rat DNA polymerase β (Pol β) was purified mainly according to Goldman and Baseman (1980) using plasmid kindly gifted by Dr. S. H. Wilson (National Institute of Environmental Health Sciences, NIH, NC, United States).

### 5′-End ^32^P-Labeling and Purification of Oligodeoxyribonucleotides

γ-[^32^P]-ATP (3000 Ci/mmol) was produced at ICBFM SB RAS. Oligodeoxyribonucleotides were 5′-[^32^P] phosphorylated with T4 polynucleotide kinase and purified by polyacrylamide 7.0 M urea gel electrophoresis as described in Evdokimov et al. (2013) and Lukyanchikova et al. (2018) followed by electroelution and precipitation with 2% solution of LiClO_4_ in acetone. The precipitated oligodeoxyribonucleotides were dissolved in 10 mM Tris-HCl (pH 8.0) and 1 mM EDTA. A NER-competent extract of CHO cells was isolated mainly according to (Reardon and Sancar, 2006).

### Protein Expression and Purification

CtXPD was cloned in the pBADM11vector and ctp44 (1–285, in the following referred to as ctp44) was cloned in the pETM11vector (EMBL-Heidelberg). Both proteins were expressed as N-terminally His-tagged proteins in *Escherichia coli* CodonPlus (DE3)-RIL cells (Stratagene). For expression, ctXPD cells were grown in TB medium at 37°C until they reached an OD_600_ value of 1.2–1.4. Expression was initiated with the addition of 0.02% L-arabinose and performed at 15°C for 18 h.

The cells for expression of ctp44 (1–285) were grown as described for ctXPD and expression was started by adding 0.5 mM IPTG at an OD_600_ value of 1.1–1.2 and performed at 15°C for 18 h.

CtXPD and ctp44 were purified using the chromatographic procedures described previously (Kuper et al., 2014) with several modifications. For the ctXPD purification, all buffers were degassed with argon. Metal affinity chromatography (HisTrap HP column, GE Healthcare) was performed followed by size exclusion chromatography (Sephacryl 16/60, GE Healthcare) (20 mM HEPES pH 7.5, 150 mM NaCl) and an additional anion exchange chromatography (AEC) step in the case of ctXPD. AEC was performed using a MonoQ 5/50 GL column (GE Healthcare) with 20 mM HEPES, pH 7.5, 50 mM NaCl, and 1 mM TCEP as loading buffer. The same buffer containing 1 M NaCl was used for gradient elution (0–50%) using 40 column volumes. The final buffer after AEC contained 20 mM HEPES, pH 7.5, 250 mM NaCl, and 1 mM TCEP.

### Fluorescence Anisotropy Assay

DNA binding was analyzed by fluorescence anisotropy measurements using the DNA substrates shown in Supplementary Table 1SA. The assay was carried out in a buffer containing 20 mM HEPES pH 7.5, 30 mM KCl, 5 mM MgCl_2_, and 1 mM TCEP supplemented with 10 nM DNA at room temperature. CtXPD and ctp44 were used at a 1:1 stoichiometric ratio at concentrations of 5 to 1500 nM as indicated. Fluorescence was detected at an excitation wavelength of 540 nm and an emission wavelength of 590 nm with a Clariostar plate reader (BMG Labtech, Germany).

The estimations have been performed according to the formula:

$$P=1000*\frac{I||-\text{I}⊥}{I||\text{I}⊥},$$

where P is Polarization (mP), I|| is the intensity of the detected light when the excitation and emission polarization is parallel and I⊥ is the intensity of detected light when the excitation and emission polarization is perpendicular.

### *In vitro* ATPase Assay

CtXPD ATPase activity was measured with the *in vitro* ATPase assay, in which ATP consumption is coupled to the oxidation of NADH via pyruvate kinase and lactate dehydrogenase activities (Kuper et al., 2014). The activity profiles were measured at 37°C in 100 μL solution containing 1.5 U pyruvate kinase, 1.9 U lactate dehydrogenase, 2 mM phosphoenolpyruvate, and 0.15 mM NADH, 10 mM KCl, 1 mM MgCl_2_, 1 mM TCEP, and 20 mM Tris-HCl (pH 8.0). We used different damage containing DNA substrates with a 5′-overhang and a Cy3 label at the 3’-end of the translocated strand and a Dabcyl modification at the 5′-end of the opposite strand (Supplementary Table 1SB). DNA was added at a final concentration of 250 nM.

The assay was carried out at saturating concentrations of ATP (2 mM). CtXPD and ctp44 were used at a 1:1 stoichiometric ratio with final concentrations of 250 nM. The reaction was initiated by the addition of ATP. For the ctXPD activation by ctp44 increasing concentrations of ctp44 (4 to 1000 nM) were added to 250 nM ctXPD in the presence of 1 μM ssDNA substrate. The activity profiles were measured at 340 nm using a Clariostar plate reader (BMG Labtech). The initial rates were recorded and the ATP consumption was determined using the molar extinction coefficient of NADH. The measurements were carried out in triplicates with at least two different protein batches. The MARS software package (BMG Labtech) was used for the analysis of the data.

### Photo-Cross-Linking of ctXPD and ctp44 to Photoactivatable DNA Probes

Photo-cross-linking was performed in the reaction mixture (20–30 μL) that contained a photoreactive 120–250 nM 5′-[^32^P]-labeled DNA probe (Supplementary Table 1SC), 200–500 nM ctXPD, and 100–200 nM ctp44 in the buffer for modification (20 mÌ HEPES, pH 7.5, 50 mÌ KCl, 5 mM MgCl_2_, and 1 mM TCEP). The experiments were carried out both in the presence and in the absence of 2.5 mM ATP. The samples were exposed to UV irradiation (312 nm) in a BIO-LINK^®^BLX (Vilber Lourmat, France) for 10 min at 1 J/cm^2*^min. The mixtures were supplemented with 4x Laemmli loading buffer, heated to 90°C for 10 min and the products were analyzed by SDS-PAGE (Laemmli, 1970). The gels were dried and subjected to autoradiography for quantification using a Typhoon FLA 9500 (GE Healthcare) and the Quantity One software.

## Results

### Affinity of ctXPD-ctp44 for Damaged DNAs

**FIGURE 1:**
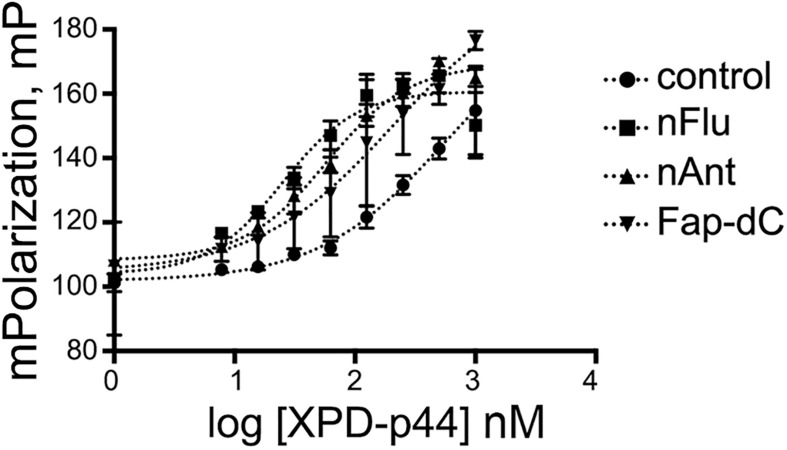
Affinity of ctXPD-ctp44 to ssDNAs containing bulky damages. Fluorescence polarization measurements were used to determine the affinity of various DNA substrates to ctXPD at increasing concentrations. Data were plotted and fitted in GraphPad and presented here as normalized values with error bars representing the SD from at least three repeats.

The quantification of the interaction of ctXPD-ctp44 with model ssDNA was obtained by fluorescence anisotropy measurements (Figure 1). The introduction of bulky damages into DNA increased the affinity of ctXPD-ctp44 for modified DNA as compared to control DNA containing no damage. The EC_50_ values are presented in Table 1. CtXPD-ctp44 exhibits an EC_50_ of 500 for undamaged DNA. A 2.5 fold affinity increase was observed for ss-Fap-dC-DNA with a EC_50_ of 200 nM. Ss-nAnt-DNA led to an almost 8-fold higher affinity with a EC_50_ of 63 nM. Noteworthy, the tightest binding with a 166-fold higher affinity (3 nM) was observed with ssDNA containing the nFlu damage.

**TABLE 1:** Characteristics of the interaction of ctXPD and ctp44 with DNAs containing bulky lesions, eliminated by NER with different efficiencies.

DNA damage	EC_50_ (ctXPD-ctp44-ssDNA), nM	ATPase activity, relative units	NER catalyzed specific excision, 137 bp dsDNA relative units
umDNA	500 ± 3	1	0
Fap-dC	200 ± 2	1.1 ± 0.2	0 *
nFlu	3 ± 1	1.3 ± 0.1	1.0**
nAnt	63 ± 1	1.4 ± 0.3	1.2**

*Maltseva et al. (2007). **Evdokimov et al. (2013).

In a separate series of experiments, we compared the binding affinity of ctXPD-ctp44 for ss-Fab(g)-dC- and ss-Fap-dC-DNA. The EC_50_ values obtained are presented in Supplementary Table 2S; the titration curves and the experiment description are presented in Supplementary Figure 3S.

### ATPase Activity of ctXPD-ctp44 Relative to Damaged DNA Substrates

**FIGURE 2:**
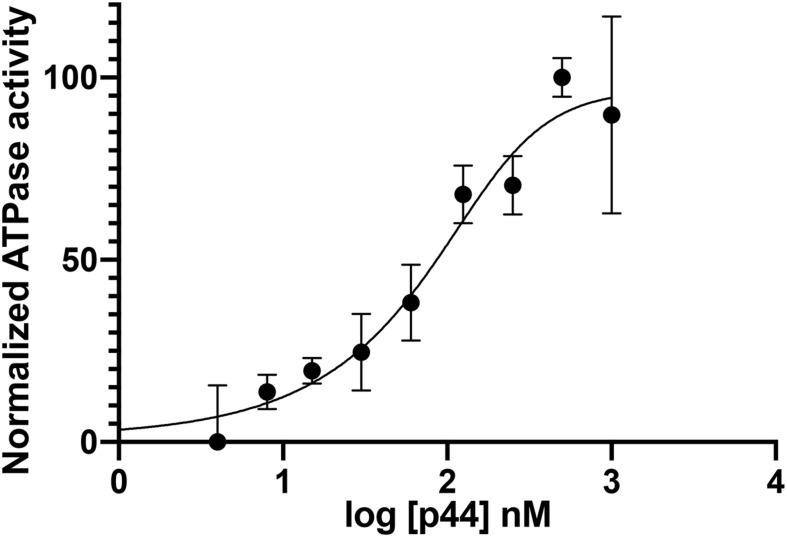
Ctp44 activation of ctXPD. Increasing amounts of ctp44 were titrated onto ctXPD resulting in ctXPD activation. The data were fitted with the GraphPad Prism software resulting in an EC_50_ of 82 nM for ctp44. Error bars represent the SD from at least three repeats. Optimal conditions were then used to perform further experiments.

To investigate the influence of the modified DNA on ctXPD activity, we performed ATPase studies with the modified DNA as substrate. To ensure that under the investigated conditions ctXPD and ctp44 form a productive complex, we titrated increasing amounts of ctp44 onto 250 nM ctXPD in the presence of 1 μM DNA. The resulting activation curve yielded an EC_50_ of 82 nM and full ctXPD activation at equimolar conditions indicating full complex formation (Figure 2). We therefore performed the subsequent experiments using equimolar concentrations of ctp44 and ctXPD. Interestingly, the ATPase activity of ctXPD-ctp44 was only slightly increased in the presence of damaged DNA compared to undamaged DNA (Table 1). This indicates that ATPase activity is not negatively affected by DNA damage. The minor change in activation depending on the used substrate could indicate that XPDs ATPase might be further stimulated by the modified DNA which would be in line with the enhanced binding of the substrate.

### Affinity Modification of ctXPD and ctp44 Proteins Using Photoactivatable DNA Probes

To select reaction conditions for the photo-cross-linking experiments, we first examined ctXPD binding to modified and unmodified ss- and ds-DNAs by EMSA (Supplementary Figure 2S). Based on the data obtained, the range of ctXPD concentrations was selected for affinity modification experiments.

**FIGURE 3:**
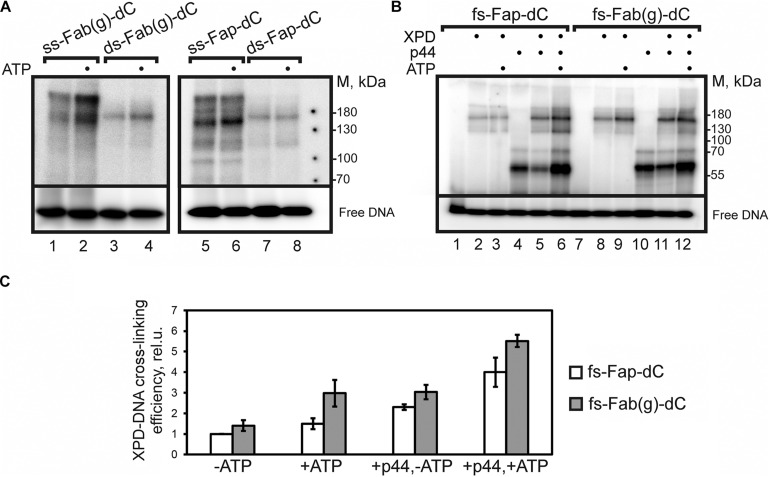
Photo-cross-linking of DNA probes to ctXPD and ctp44. **(A)** *Autoradiogram of a SDS-PAGE gel after ctXPD cross-linking product separation. ss- or ds-Fab(g)- dC-, ss- or ds-Fap-dC-DNA were used as DNA probes. Reaction mixtures containing 200 nM ctXPD, 100 nM ss-Fab(g)-dC (lanes 1, 2), ds-Fab(g)-dC (lanes 3, 4), ss-Fap-dC (lanes 5, 6) or ds-Fap-dC (lanes 7, 8), 2.5 mM ATP (lanes 2, 4, 6, 8)*, *and buffer components were analyzed by electrophoresis in a 10% polyacrylamide gel.* **(B)** *Autoradiogram of a SDS-PAGE gel after ctXPD/ctp44 cross-linking products separation. fs-Fap-dC- or fs-Fab(g)-dC-DNA were used as DNA probes. Reaction mixtures contained 120 nM fs-Fap-dC- (lanes 1–6) or fs-Fab(g)-dC-DNA probe (lanes 7–12), 200 nM ctXPD (lanes 2, 3, 5, 6, 8, 9, 11, 12), 200 nM ctp44 (lanes 4–6, 10–12) and buffer components. The experiments were carried out both in the presence (lanes 3, 6, 9, 12) and absence of 2.5 mM ATP. The samples were analyzed by electrophoresis in a 10% polyacrylamide gel.* **(C)** The dependence of photo-cross-linking efficiency of ctXPD on the type of DNA probe and the presence/absence of ctp44 and ATP in the reaction mixture. The efficiency of ctXPD cross-linking to fs-Fap-dC-DNA in the absence of ctp44 and ATP was taken as 1. Error bars represent the SD from triplicate measurements.

We compared the cross-linking efficiency of ctXPD to DNA probes containing the unrepairable and repairable photoactivatable damages, i.e., Fap-dC and Fab(g)-dC, respectively (Figure 3A). The photo-cross-linking of ctXPD to ss- and ds-DNA demonstrated that the products of ctXPD-DNA photo-cross-linking were formed three times more efficiently when the ssDNA probes were used. A higher yield of cross-linked ctXPD when using a single-stranded DNA probe is a rather expected result, since the higher affinity of ctXPD for single-stranded DNA is accompanied by a longer residence time of the protein on the ssDNA probe. Next, we analyzed the cross-linking of the proteins with fs-Fap-dC- and fs-Fab(g)-dC-DNA probes with a 5′-overhang (16 nt) and ds fragment (38 bp) containing the photoactivatable damages (fs designates “fork structure”). The [^32^P]-label was attached to the 5′-end of the ss 5′-overhang.

Under the conditions used, ctXPD formed a major covalent adduct with the fs probes, which displayed the same electrophoretic mobility both in the presence and in the absence of ATP and ctp44 (Figure 3B). In all the experiments DNA bearing the repairable Fab(g)-dC were more effective as cross-linking probes. The level of ctXPD cross-linking was influenced by the presence of ATP and ctp44.

The minimal levels of ctXPD cross-linking with fs DNA probes in the absence of both ATP and ctp44 (Figure 3B, lanes 2, 8; 3C) presumably results from ctXPD interaction with photoactivatable damages located in the double stranded area of the DNAs. Addition of ATP to ctXPD increased the level of the ctXPD adducts formed with fs-Fab(g)-DNA by a factor of 2.1 but did not enhance ctXPD adduction with fs-Fap-dC-DNA (Figure 3B, lanes 3, 9; 3C). Addition of ATP to ctXPD-ctp44 promoted the cross-linking of ctXPD to fs-Fap-dC-DNA (by a factor of 2.6) and, to a lesser extent, to fs-Fab(g)-dC (by a factor of 1.8) (Figure 3B, lanes 5, 6, 11, 12; 3C).

Our analysis also showed, that the ctp44 protein formed adducts with both photoactivatable fs-DNAs with high efficiency in the absence of ctXPD and ATP in the reaction mixtures. The presence of ctXPD decreased the photo-cross-linking of ctp44 in the absence of ATP. However, in the presence of ATP and ctXPD, the efficiency of the cross-linking of ctp44 increased by a factor of about 2.0 compared to that in the mixture without ATP (Figure 3B, lanes 5–6, 11–12).

## Discussion

The NER system exhibits an extremely broad substrate specificity, wherein the efficiency of the damage removal varies by orders of magnitude. The study of the relationship between the structure of the damages and the rate of its elimination is important both for the prevention of accelerated accumulation of mutations in the cells and the development of methods to increase the effectiveness of chemotherapy or complex cancer therapy by the accumulation of induced DNA damages (Kiwerska and Szyfter, 2019).

In this work, we studied the efficiency of protein-DNA interaction utilizing a series of different bulky DNA damages and the ctXPD-ctp44 protein complex from *C. thermophilum* (Kuper et al., 2014). XPD is thought to be the main sensor for the verification of bulky damages. However, the important role in the damage verification process is distributed between the subunits of core TFIIH including p44, which directly interacts with XPD and modulates its activity (Coin et al., 1998; Kuper et al., 2014).

A comparison of the binding of ctXPD-ctp44 to ssDNAs that contained the Fap-dC, nAnt, and nFlu damages to unmodified DNA (umDNA) showed that the affinity of ctXPD-ctp44 for these DNAs (Table 1) increased as follows: umDNA < Fap-dC-DNA < nAnt-DNA < < nFlu-DNA. These results correlate well with the reported removal of these damages within the NER cascade. Recently it has been shown that the efficiency of the removal of Fab(g)-dC from DNA was about half of that observed for the nFlu and nAnt damages (Lukyanchikova et al., 2016), and the Fap-dC damage was almost unrepaired (excision efficiency increased in a row: umDNA≈Fap-dC-DNA < < nFlu-DNA < nAnt-DNA (Evdokimov et al., 2011, 2018). The NER system removes the bulky nFlu and nAnt damages from DNA with almost the same high efficiency. However, the data obtained in this work have shown that the affinity of ctXPD-ctp44 for ss-nFlu-DNA (EC_50_≈3 nM) is much higher than for ss-nAnt-DNA (EC_50_≈60 nM). According to the results of computer simulations, the presence of the Flu moiety in dsDNA can lead to several changes in the DNA structure, which reduce the likelihood of a productive XPC-DNA complex formation (Evdokimov et al., 2018).

Thus, it can be assumed that good substrate properties of nFlu-containing DNA in the NER process are determined by the high affinity of ctXPD-ctp44 for DNA containing this damage. Despite the fact that the ATPase activity of ctXPD-ctp44 was increased in the presence of damaged DNA compared to undamaged DNA, the observed differences are small. In the case of ssDNA induced ATPase activities the small changes that we observed could hint at different ATPase activation, but the major information regarding specificity should be inferred from the binding data.

Affinity cross-linking of ctXPD and ctp44 with DNA probes of different structures containing the photoactivatable damages revealed different interaction capabilities of these proteins with Fap-dC-DNA and Fab(g)-dC-DNA. The results on the photo-cross-linking of ctXPD to ss- and ds-Fap-dC-DNA correlate well with the data on the higher affinity of ctXPD to ssDNA (Kuper et al., 2014). The increased affinity permits a longer residence time of XPD on the ssDNA probe, which promotes the formation of covalent adducts. In contrast to unrepaired Fap-dC, the Fab(g)-dC damage is removed from DNA quite effectively (Evdokimov et al., 2011). The simultaneous presence of ATP and ctp44, which promotes the activity of ctXPD by about an order of magnitude (Kuper et al., 2014), levels the difference between the yields of ctXPD adduction. The increase of the proteins photo-cross-linking level may result from specific structural changes in the ctXPD-ctp44 complex.

Our results confirm the important role of p44 in the verification of damaged DNA by XPD. These results indicate that p44 interacts with damaged DNAs. This assumption is partially supported by the recently published data (Barnett et al., 2019) on the interaction of the ð44–p62 heterodimer with DNA and will be the subject of further research.

## Data Availability Statement

The original contributions presented in the study are included in the article/Supplementary Material, further inquiries can be directed to the corresponding author.

## Author Contributions

IP, JoK, JeK, NN, and RA conducted the experiments. IP, JoK, and RA designed the model DNA structures. IP, JoK, and CK conceived the project. IP, NN, JoK, CK, and OL analyzed the data and wrote the manuscript. All authors contributed to the article and approved the submitted version.

## Conflict of Interest

The authors declare that the research was conducted in the absence of any commercial or financial relationships that could be construed as a potential conflict of interest. The handling editor declared a shared affiliation and a past co-authorship with one of the authors OL at the time of review.
