# Supplementary figures and images for: The Interaction Efficiency of XPD-p44 With Bulky DNA Damages Depends on the Structure of the Damage
Source: Front Cell Dev Biol. 2021 Mar 11;9:617160. doi: 10.3389/fcell.2021.617160 (PMC7991749; doi:10.3389/fcell.2021.617160)

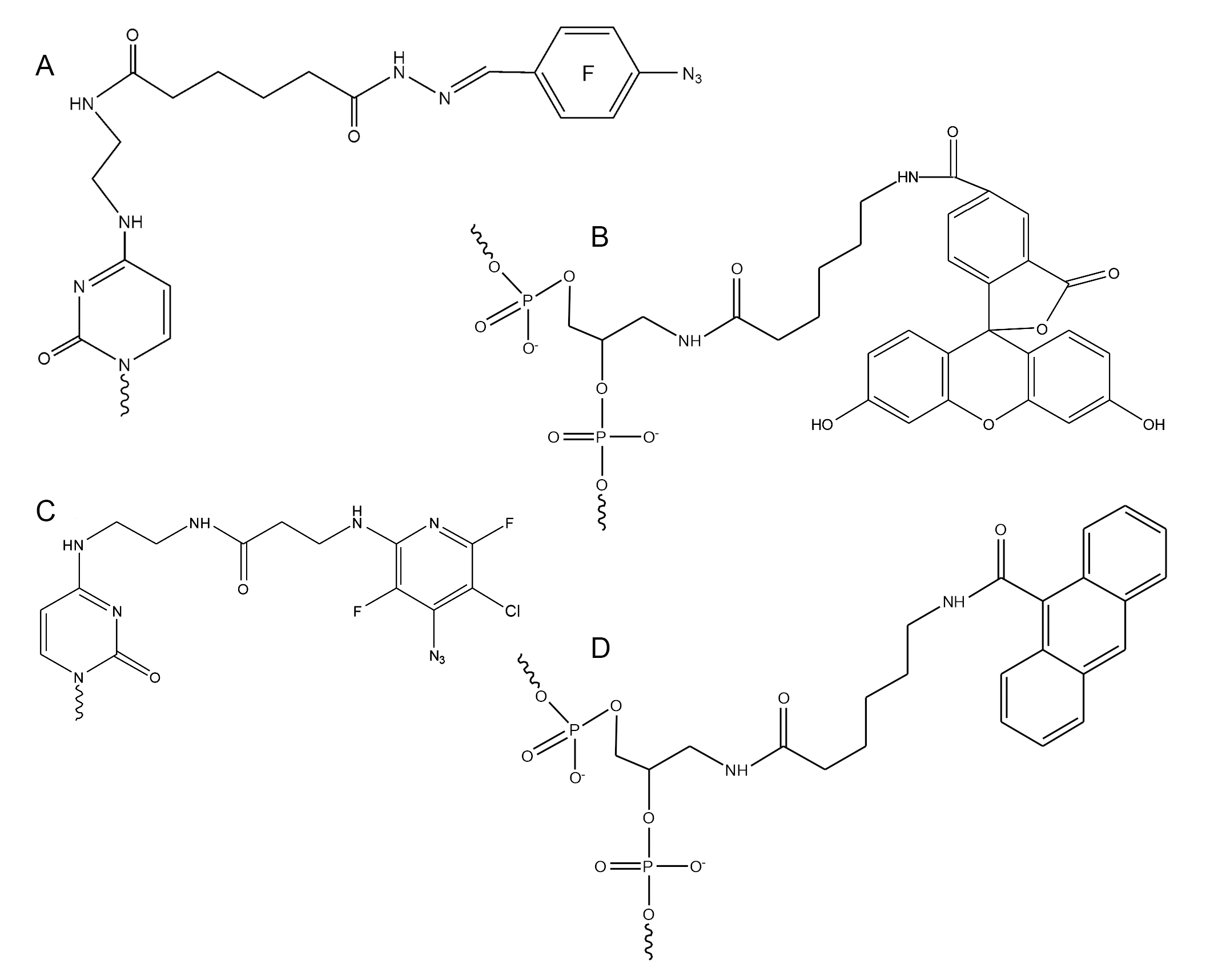

Supplement: Supplementary file 1 [file Image_1.TIF]

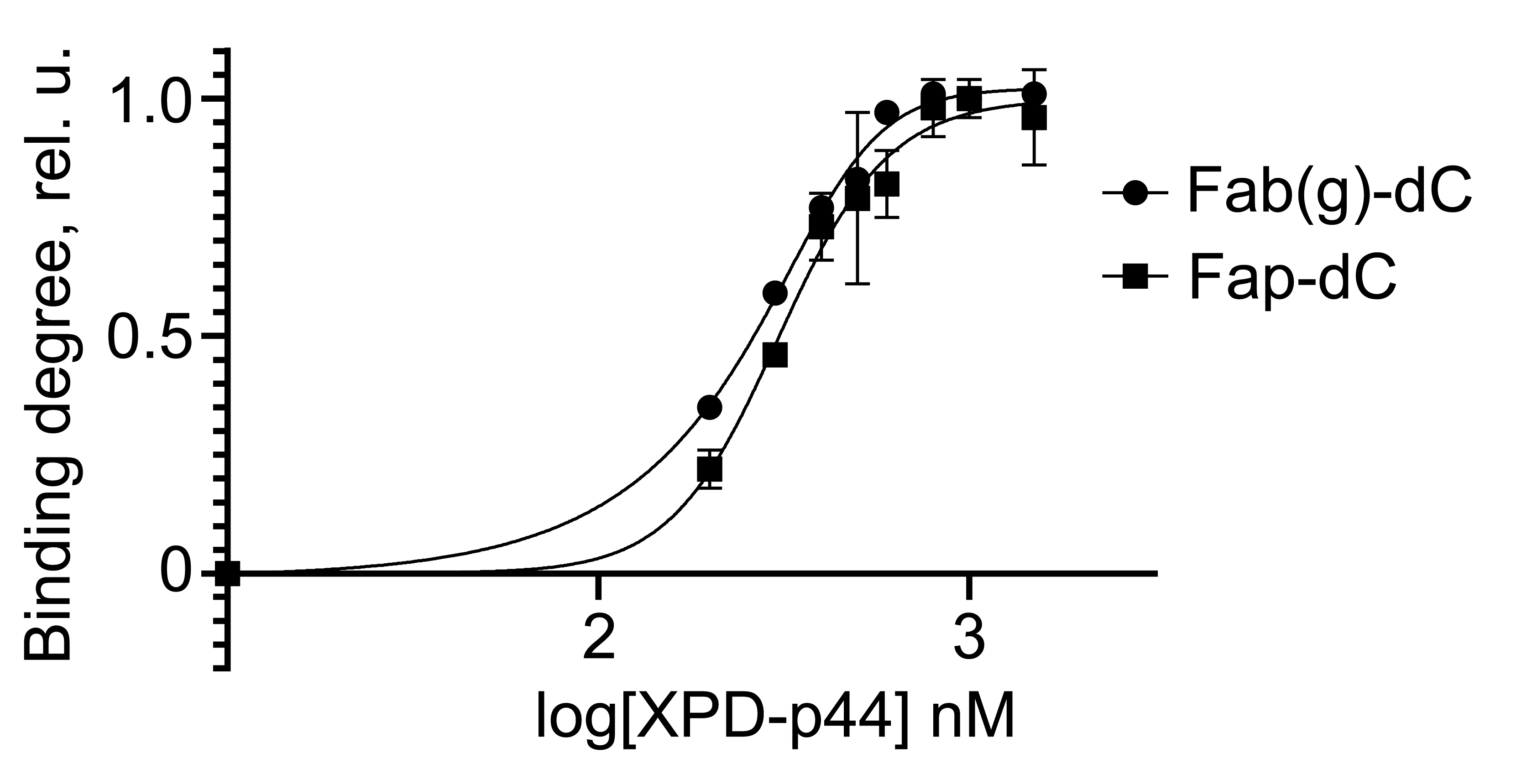

Supplement: Supplementary file 3 [file Image_3.TIF]
