# Supplementary material for: The Interaction Efficiency of XPD-p44 With Bulky DNA Damages Depends on the Structure of the Damage
Source: Front Cell Dev Biol. 2021 Mar 11;9:617160. doi: 10.3389/fcell.2021.617160 (PMC7991749; doi:10.3389/fcell.2021.617160)
Supplement: Supplementary file 4 [file Table_1.DOCX]

## Supplementary Table 1S. Sequences of DNA substrates used in the study.

| **Name** | **Length, nt** | **Sequence** |
| --- | --- | --- |
| **A. DNAs for fluorescence anisotropy measurements** | | |
| ss-um | 49 | 5'-ATGCCTACAGCAAGCCTATGCCTACAGCATCCAGGGCGACGGTGCCGAG-Cy3 |
| ss-Ant | 49 | 5'-ATGCCTACAGCAAGCCTATGCCTACAGCATCCAGGG**A**GACGGTGCCGAG-Cy3 |
| ss-nFlu | 49 | 5'-ATGCCTACAGCAAGCCTATGCCTACAGCATCCAGGG**F**GACGGTGCCGAG-Cy3 |
| ss-Fap-dC | 49 | 5'-ATGCCTACAGCAAGCCTATGCCTACAGCATCCAGGG**C**GACGGTGCCGAG-Cy3 |
| ss-Fab(g)-dC | 49 | 5'-ATGCCTACAGCAAGCCTATGCCTACAGCATCCAGGG**B**GACGGTGCCGAG-Cy3 |
| **B. DNAs for ATPase activity** **assay** | | |
| fs-um | 49/23 | 5'-ATGCCTACAGCAAGCCTATGCCTACAGCATCCAGGGCGACGGTGCCGAG-Cy3  \|\|\|\|\|\|\|\|\|\|\|\|\|\|\|\|\|\|\|\|\|\|\|  3' CGTAGGTCCCGCTGCCACGGCTC-Dab |
| fs-Fap-dC | 49/23 | 5'-ATGCCTACAGCAAGCCTATGCCTACAGCATCCAGGG**C**GACGGTGCCGAG-Cy3  \|\|\|\|\|\|\|\|\|\|\|\|\|\|\|\|\|\|\|\|\|\|\|  3' CGTAGGTCCCGCTGCCACGGCTC-Dab |
| fs-nAnt | 49/23 | 5'-ATGCCTACAGCAAGCCTATGCCTACAGCATCCAGGG**A**GACGGTGCCGAG-Cy3  \|\|\|\|\|\|\|\|\|\|\|\|\|\|\|\|\|\|\|\|\|\|\|  3' CGTAGGTCCCGCTGCCACGGCTC-Dab |
| fs-nFlu | 49/23 | 5'-ATGCCTACAGCAAGCCTATGCCTACAGCATCCAGGG**F**GACGGTGCCGAG-Cy3  \|\|\|\|\|\|\|\|\|\|\|\|\|\|\|\|\|\|\|\|\|\|\|  3' CGTAGGTCCCGCTGCCACGGCTC-Dab |
| **C. DNA probes for affinity modification and EMSA** | | |
| ss-Fap-dC | 54 | 5'-AAGCCTATGCCTACAGCATCCAGGG**C**GACGGTGCCGAGGATGACGATGAGCGCA |
| fs-Fap-dC | 54/38 | 5'-AAGCCTATGCCTACAGCATCCAGGG**C**GACGGTGCCGAGGATGACGATGAGCGCA  \|\|\|\|\|\|\|\|\|\|\|\|\|\|\|\|\|\|\|\|\|\|\|\|\|\|\|\|\|\|\|\|\|\|\|\|\|\|  3' GTAGGTCCCGCTGCCACGGCTCCTACTGCTACTCGCGT |
| ds-Fap-dC | 54/54 | 5'-AAGCCTATGCCTACAGCATCCAGGG**C**GACGGTGCCGAGGATGACGATGAGCGCA  \|\|\|\|\|\|\|\|\|\|\|\|\|\|\|\|\|\|\|\|\|\|\|\|\|\|\|\|\|\|\|\|\|\|\|\|\|\|\|\|\|\|\|\|\|\|\|\|\|\|\|\|\|\|  3'-TTCGGATACGGATGTCGTAGGTCCCGCTGCCACGGCTCCTACTGCTACTCGCGT |
| ss-Fab(g)-dC | 54 | 5'-AAGCCTATGCCTACAGCATCCAGGG**B**GACGGTGCCGAGGATGACGATGAGCGCA |
| fs-Fab(g)-dC | 54/38 | 5'-AAGCCTATGCCTACAGCATCCAGGG**B**GACGGTGCCGAGGATGACGATGAGCGCA  \|\|\|\|\|\|\|\|\|\|\|\|\|\|\|\|\|\|\|\|\|\|\|\|\|\|\|\|\|\|\|\|\|\|\|\|\|\|  3' GTAGGTCCCGCTGCCACGGCTCCTACTGCTACTCGCGT |
| ds-Fab(g)-dC | 54/54 | 5'-AAGCCTATGCCTACAGCATCCAGGG**B**GACGGTGCCGAGGATGACGATGAGCGCA  \|\|\|\|\|\|\|\|\|\|\|\|\|\|\|\|\|\|\|\|\|\|\|\|\|\|\|\|\|\|\|\|\|\|\|\|\|\|\|\|\|\|\|\|\|\|\|\|\|\|\|\|\|\|  3'-TTCGGATACGGATGTCGTAGGTCCCGCTGCCACGGCTCCTACTGCTACTCGCGT |
| ss-um | 54 | 5'-AAGCCTATGCCTACAGCATCCAGGGCGACGGTGCCGAGGATGACGATGAGCGCA |
| ds-um | 54/54 | 5'-AAGCCTATGCCTACAGCATCCAGGGCGACGGTGCCGAGGATGACGATGAGCGCA  \|\|\|\|\|\|\|\|\|\|\|\|\|\|\|\|\|\|\|\|\|\|\|\|\|\|\|\|\|\|\|\|\|\|\|\|\|\|\|\|\|\|\|\|\|\|\|\|\|\|\|\|\|\|  3'-TTCGGATACGGATGTCGTAGGTCCCGCTGCCACGGCTCCTACTGCTACTCGCGT |

**Designations in the table:
A –** nAnt**, F –** nFlu**, C -** Fap-dC, **B –** Fab(g)-dC

## Table 2S. Binding affinities of the ctXPD-ctp44 complex to ssDNAs containing bulky damages.

| **DNA damage** | **EC_50_ (ctXPD-ctp44-ssDNA), nM** |
| --- | --- |
| Fap-dC | 307±1.1 |
| Fab(g)-dC | 268±1.0 |
