# Supplementary material for: The Interaction Efficiency of XPD-p44 With Bulky DNA Damages Depends on the Structure of the Damage
Source: Front Cell Dev Biol. 2021 Mar 11;9:617160. doi: 10.3389/fcell.2021.617160 (PMC7991749; doi:10.3389/fcell.2021.617160)
Supplement: Supplementary file 5 [file Data_Sheet_1.PDF]

Supplementary Material

Supplementary Figure 1S. Structures of the model lesions.

- **(A)** Fab(g)-dC – exo-N-[4-(4-azido-2,3,5,6-tetrafluorobenzylidenehydrazinocarbonyl)-butylcarbamoyl]-2′-deoxycytidine.
- **(B)** nFlu – (N-[6-(dipivaloyl-5(6)-fluoresceinylcarbamoyl)hexanoyl]-O1-(4,4′dimethoxytrityl)-O2-[(diisopropylamino)(2-cyanoethoxy)phosphino]-3-amino-1,2-propanediol).
- **(C)** Fap-dC – exo-N-{2-[N-(4-azido-2,5-difluoro-3-chloropyridin-6-yl)-3-aminopropionyl]aminoethyl}-deoxycytidinin.
- **(D)** nAnt – N-[6-(9-anthracenyl-carbamoyl)hexanoyl]-3-amino-1,2-propanediol.

Supplementary Figure 2S. EMSA studies for the analysis of ctXPD-DNA binding.

(A-D) EMSA of ds-Fab(g)-DNA (A), ds-umDNA (B), ss-Fab(g)-DNA (C), ss-umDNA (D) binding by ctXPD.

Reaction mixtures containing 100 nM 5'-[^{32}P]-labeled DNA (table 1S-C), ctXPD (0, 25, 50, 100, 150, 250, 500 and 750 nM) and buffer components (20 mM Tris, pH 7.4, 50 mM KCl, 1 mM DTT, 0.1 mg/mL BSA, 0.1 mM EDTA, 0.05% NP-40, and 8% glycerol) were incubated for 15 min at 30°C. The protein-DNA complexes were stabilized by the addition of 1 μL of 5% glutaraldehyde and incubation at 30°C for 3 min before the EMSA analysis. The samples were loaded on a 6% PAGE gel (aa:bis = 40 : 1) in 1X TBE buffer at 4°C. The standard gel analysis was applied. DNA-XPD complexes are marked.

(E) Quantitative analysis of fractions of DNA molecules in complexes with ctXPD.

The results of EMSA were quantified and plotted. Error bars represent the SD from triplicate measurements.

Supplementary Figure 3S. Binding affinity of the ctXPD-ctp44 complex to ssDNAs containing bulky damages.

Fluorescence polarization measurements were used to determine the affinity of ctXPD-ctp44 to ss-Fab(g)-dC- and ss-Fap-dC-DNA. The measurements were carried out as described in the ‘Materials and methods’ section; the mixtures also contained 2 mM ATP. Error bars represent SD from triplicate measurements.
